# Neonatal mortality associated with perinatal asphyxia: a population-based study in a middle-income country

**DOI:** 10.1186/s12884-021-03652-5

**Published:** 2021-02-27

**Authors:** Mandira D. Kawakami, Adriana Sanudo, Mônica L. P. Teixeira, Solange Andreoni, Josiane Q. X. de Castro, Bernadette Waldvogel, Ruth Guinsburg, Maria Fernanda de Almeida

**Affiliations:** 1 Escola Paulista de Medicina, Universidade Federal de São Paulo, Rua Los Angeles, 40, São Paulo, CEP 04564-030 Brazil; 2 Fundação Sistema Estadual de Análise de Dados, São Paulo, Brazil

**Keywords:** Infant newborn, Perinatal asphyxia, Neonatal mortality, Developing countries, Epidemiological studies

## Abstract

**Background:**

It is challenging to decrease neonatal mortality in middle-income countries, where perinatal asphyxia is an important cause of death. This study aims to analyze the annual trend of neonatal mortality with perinatal asphyxia according to gestational age in São Paulo State, Brazil, during a 10-year period and to verify demographic, maternal and neonatal characteristics associated with these deaths.

**Methods:**

Population-based study of neonatal deaths associated with perinatal asphyxia from 0 to 27 days in São Paulo State, Brazil, from 2004 to 2013. Perinatal asphyxia was considered as associated to death if intrauterine hypoxia, birth asphyxia or neonatal aspiration of meconium were noted in any line of the Death Certificate according to ICD-10. Poisson Regression was applied to analyze the annual trend of neonatal mortality rate according to gestational age. Kaplan-Meier curve was used to assess age at death during the 10-year study period. Hazard ratio of death during the neonatal period according to gestational age was analyzed by Cox regression adjusted by year of birth and selected epidemiological factors.

**Results:**

Among 74,002 infant deaths in São Paulo State, 6648 (9%) neonatal deaths with perinatal asphyxia were studied. Neonatal mortality rate with perinatal asphyxia fell from 1.38‰ in 2004 to 0.95‰ in 2013 (*p* = 0.002). Reduction started in 2008 for neonates with 32–41 weeks, in 2009 for 28–31 weeks, and in 2011 for 22–27 weeks. Median time until 50% of deaths occurred was 25.3 h (95%CI: 24.0; 27.2). Variables independently associated with higher risk of death were < 7 prenatal visits, 1st minute Apgar score 0–3, and death at the same place of birth. Cesarean delivery compared to vaginal was protective against death with perinatal asphyxia for infants at 28–36 weeks.

**Conclusions:**

There was an expressive reduction in neonatal mortality rates associated with perinatal asphyxia during this 10-year period in São Paulo State, Brazil. Variables associated with these deaths highlight the need of public health policies to improve quality of regionalized perinatal care.

## Background

The commitment to the Millennium Development Goals (MDG) have catalyzed attention and investments in 195 countries across the world since 2000 and MDG 4 was the one dedicated to the reduction of under-5 mortality by two-thirds by 2015. In this period (1990–2015), while the number of under-5 deaths decreased by 52% due to strategies assigned to preventable diseases like infection and nutritional deficiencies, the neonatal mortality remained a big challenge [1]. Two of the three leading causes of under-5 child mortality are neonatal, especially preterm birth complications (17.8%) and intrapartum-related events (11.6%) [2]. Neonatal deaths are predominant in the first week after birth, related to maternal health care before and after the time of birth. Intrapartum-related neonatal deaths are also associated with significant morbidity, resulting in a burden of 42 million disability adjusted life years [3].

Brazil is a middle-income country whose economic growth and investments in health care during the last decade allowed a reduction in child mortality determined by MDG 4 before the deadline. In 2011, the neonatal mortality rate (NMR) was 10.6 out of 15.3 infant deaths per 1000 live births [4]. Neonatal deaths in Brazil are due to prematurity, perinatal asphyxia and congenital malformations [5]. The rates of early neonatal deaths associated with perinatal asphyxia of infants with birth weight ≥ 2500 g had a significant reduction from 0.81 per 1000 live births in 2005 to 0.65 in 2010, however these figures of preventable deaths were heterogeneous among the five Brazilian regions [6].

The Southeast Region is one of the most developed in Brazil, with a great contribution from São Paulo State, responsible for 32% of the national income, with a Human Development Index (HDI) of 0.783 in 2010 and listed as the 19th largest economy of the world [7, 8]. Infant mortality rate was 11.47‰ in São Paulo State in 2013 and the NMR of 7.85‰ was one of the lowest in the country [9]. Despite the high prenatal coverage, with 91% of the live births with four or more antenatal visits and almost 100% of hospital births, perinatal asphyxia and meconium aspiration syndrome still accounted for 22% of the early neonatal deaths in São Paulo State during 2001–2003 [10].

The great challenge to achieve the “Sustainable Development Goals” until 2035 leads us to the bottleneck of perinatal health assistance. In this context, the objectives of this study were to analyze the annual trend of neonatal mortality with perinatal asphyxia according to gestational age (GA) in São Paulo State, to evaluate the annual risk of death with perinatal asphyxia and to verify demographic, maternal, and neonatal characteristics associated with these deaths.

## Method

This is a population-based study of neonatal deaths associated with perinatal asphyxia from 0 to 27 days from 2004 to 2013 in São Paulo State, Southeast Brazil. Perinatal asphyxia was defined if any of the following codes of the 10th revision of International Classification of Diseases (ICD 10) [11] were noted in any line of the Death Certificate: intrauterine hypoxia (P20.0; P20.1; P20.9), birth asphyxia (P21.0; P21.1; P21.9) or neonatal aspiration of meconium (P24.0). Exclusion criteria were birth weight < 500 g, GA < 22 weeks, congenital malformation, or death in São Paulo State with birth outside the State.

As referred in a previous study [12], the database of neonatal deaths with perinatal asphyxia was extracted from the vital statistics database provided by the Foundation of the State System of Data Analysis (Fundação SEADE) that covers 99.7 and 99.8% of births and deaths, respectively, in the State [13].

The following data were analyzed: 1) Maternal data: age, marital status, schooling, children born alive and dead in previous pregnancies, prenatal visits, single or multiple pregnancy and delivery mode; 2) Birth characteristics: birth at mother’s home municipality, death at the same municipality of birth, death at the same place of birth, place of birth and death, day of the week and time of birth; 3) Neonatal data: gestational age (weeks), date and time of birth, sex, Apgar scores, ethnicity, birth weight (grams), congenital malformations, time and date of death, age of death and causes mentioned in all lines of the Death Certificate.

Poisson Regression Model with robust variance was applied to analyze the annual trend of NMR according to GA. Epidemiological analysis of deaths by year of event was done with chi-square test for linear trend. Kaplan-Meier curve was used to assess age at death during the 10-year study period. Hazard ratio of death during the neonatal period according to gestational age was analyzed by Cox regression adjusted by year of birth and selected epidemiological factors.

Statistical analysis was done using Stata/SE 14.2 (StataCorp, 2017. College Station, TX: StataCorp LLC). The study was approved by the Ethics Committee on Human Research at Universidade Federal de São Paulo (Approval 451.644), and by the Board of Directors of Fundação SEADE.

## Results

From 2004 to 2013, there were 6,097,692 live births in São Paulo State with 74,002 deaths in the first year after birth, resulting in an infant mortality rate of 12.13 deaths per 1000 live births [14]. Of these infant deaths, 8725 (11.8%) occurred within 0–27 days with one of the ICD-10 perinatal asphyxia codes noted in any line of the Death Certificate. Among them, 576 had birth weight < 500 g and/or GA < 22 weeks, 1407 had congenital malformations and 94 unknown GA, resulting in 2077 (23.8%) exclusions. Therefore, 6648 neonatal deaths with perinatal asphyxia were studied.

**Fig. 1:**
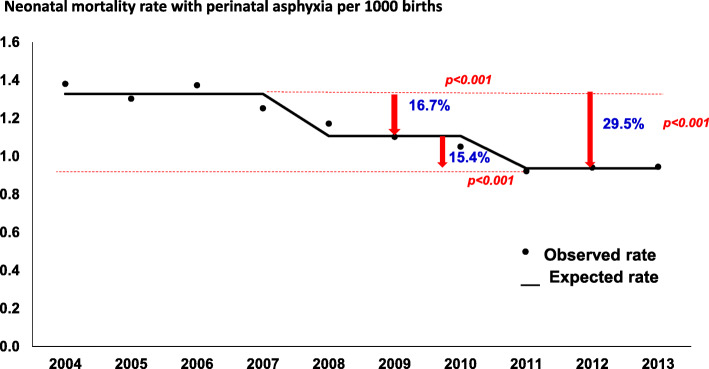
Annual trend of observed and expected neonatal mortality rates with perinatal asphyxia adjusted by Poisson regression analysis, São Paulo State, Brazil: 2004–2013

Figure 1 shows the annual trend of mortality rate with perinatal asphyxia adjusted by Poisson Regression analysis, ranging from 1.38‰ in 2004 to 0.95‰ in 2013, which represents a decrease of 32%, that started in 2008 (*p* = 0.002). For all GA groups there was a reduction in asphyxia associated neonatal mortality rate (Poisson Regression Analysis: *p* < 0.0001). The decrease of NMR during the study period according to GA was 28, 39, 63, 43, and 80%, respectively, for infants with 22–27 weeks, 28–31 weeks, 32–36 weeks, 37–41 weeks, and ≥ 42 weeks of GA **(**Table 1).

**Table 1:** Neonatal mortality rate with perinatal asphyxia per 1000 live births according to year of birth and gestational age, São Paulo State, Brazil: 2004–2013

Year of birth	Total			22–27 weeks			28–31 weeks			32–36 weeks			37–41 weeks			≥42 weeks		
	Asphyxia Deaths	Live Births*	Rate (‰)	Asphyxia Deaths	Live Births*	Rate (‰)	Asphyxia Deaths	Live Births*	Rate (‰)	Asphyxia Deaths	Live Births*	Rate (‰)	Asphyxia Deaths	Live Births *	Rate (‰)	Asphyxia Deaths	Live Birth*	Rate (‰)
**2004**	849	614,766	**1.38**	235	2414	**97.35**	134	4902	**27.34**	138	38,954	**3.54**	336	564,519	**0.60**	6	3977	**1.51**
**2005**	794	609,396	**1.30**	262	2543	**103.03**	103	4781	**21.54**	116	39,399	**2.94**	307	558,661	**0.55**	6	4012	**1.50**
**2006**	722	525,561	**1.37**	220	2267	**97.05**	121	4278	**28.28**	96	35,728	**2.69**	280	480,261	**0.58**	5	3027	**1.65**
**2007**	687	548,431	**1.25**	192	2238	**85.79**	110	4394	**25.03**	108	37,714	**2.87**	268	501,662	**0.53**	9	2423	**3.71**
**2008**	640	545,953	**1.17**	212	2244	**94.47**	96	4318	**22.23**	90	37,670	**2.39**	236	499,492	**0.47**	6	2229	**2.69**
**2009**	646	586,030	**1.10**	207	2572	**80.48**	103	4961	**20.76**	81	41,763	**1.94**	253	534,489	**0.47**	2	2245	**0.89**
**2010**	624	593,711	**1.05**	215	2513	**85.56**	91	4743	**19.19**	88	43,077	**2.04**	226	541,260	**0.42**	4	2118	**1.89**
**2011**	553	599,928	**0.92**	165	2503	**65.92**	94	5404	**17.40**	86	44,858	**1.92**	204	541,613	**0.38**	4	5550	**0.72**
**2012**	566	601,285	**0.94**	181	2354	**76.89**	85	7439	**11.43**	93	55,494	**1.68**	197	518,926	**0.38**	10	17,072	**0.59**
**2013**	567	599,953	**0.95**	203	2917	**69.59**	101	6065	**16.65**	83	64,077	**1.30**	177	517,049	**0.34**	3	9845	**0.31**

*Live Births - Source: *Fundação* SEADE

Table 2 shows that the reduction of neonatal deaths with asphyxia starts in 2008 for moderate preterm (32–36 weeks) and term (37–41 weeks) neonates, in 2009 for the very preterm (28–31 weeks), and more recently, in 2011, for the extremely preterm neonates (22–27 weeks)**.**

**Table 2:** Hazard ratio (95% confidence interval) of neonatal mortality rate with perinatal asphyxia according to year of birth and gestational age, considering the year of 2004 as a reference for each gestational age strata. São Paulo State, Brazil: 2004–2013

	Total	22–27 weeks	28–31 weeks	32–36 weeks	37–41 weeks
**2004**	1 (reference)	1 (reference)	1 (reference)	1 (reference)	1 (reference)
**2005**	0.943 (0.856–1.039)	1.058 (0.887–1.262)	0.788 (0.610–1.019)	0.831 (0.649–1.064)	0.923 (0.790–1.078)
**2006**	0.995 (0.901–1.098)	0.997 (0.829–1.198)	1.034 (0.809–1.323)	0.758 (0.584–0.984)	0.979 (0.836–1.148)
**2007**	0.907 (0.820–1.003)	0.881 (0.728–1.066)	0.916 (0.712–1.178)	0.808 (0.628–1.040)	0.898 (0.764–1.054)
**2008**	0.849 (0.766–0.940)	0.970 (0.806–1.168)	0.813 (0.626–1.057)	0.674 (0.517–0.879)	0.794 (0.672–0.937)
**2009**	0.798 (0.720–0.884)	0.827 (0.685–0.996)	0.759 (0.587–0.982)	0.547 (0.416–0.720)	0.795 (0.675–0.936)
**2010**	0.761 (0.686–0.844)	0.879 (0.730–1.057)	0.702 (0.538–0.916)	0.577 (0.441–0.753)	0.710 (0.593–0.830)
**2011**	0.667 (0.600–0.743)	0.677 (0.555–0.826)	0.636 (0.489–0.828)	0.541 (0.413–0.708)	0.633 (0.532–0.753)
**2012**	0.682 (0.613–0.758)	0.790 (0.651–0.959)	0.418 (0.318–0.548)	0.473 (0.363–0.615)	0.638 (0.535–0.760)
**2013**	0.684 (0.615–0.761)	0.715 (0.592–0.862)	0.609 (0.470–0.789)	0.366 (0.278–0.480)	0.575 (0.479–0.690)

Information on studied variables was available for 90% or more of the population as shown in Table 3. Throughout these 10 years of study, most neonates that died with perinatal asphyxia were born at hospitals, in the same municipality of mothers’ home, during daytime and at weekdays. Most of these hospitals were public, even though there was a decrease of births in public hospitals along this period. Most deaths occurred at the same hospital of birth, at the maternal home municipality. Of the neonatal deaths, 10% occurred during the first hour after birth. Adolescent mothers were almost 25% of the studied population, half of mothers were primiparous and had a partner, with improvement in schooling throughout the years. The majority had more than four prenatal visits. Cesarean sections increased from 49% in 2004 to 53% in 2013 in infants that died with perinatal asphyxia in the neonatal period. The proportion of term infants that died with perinatal asphyxia decreased from 40% in 2004 to 31% in 2013. Conversely, the proportion of very low birth weight infants (< 1500 g) and male neonates increased during the study period.

**Table 3:** Demographic, maternal, and neonatal characteristics of infants that died up to 27 days after birth with perinatal asphyxia in each year of the study, São Paulo State, Brazil: 2004–2013

	Ignored^a^ (%)	2004	2005	2006	2007	2008	2009	2010	2011	2012	2013	***p-value***^***#***^
		*n* = 849	*n* = 794	*n* = 722	*n* = 687	*n* = 640	*n* = 646	*n* = 624	*n* = 553	*n* = 566	*n* = 567	
**Place and time of birth**												
Birth at mother’s home municipality	0	77%	77%	78%	76%	76%	75%	77%	77%	74%	75%	0.202
Birth at hospital	0.02	98%	99%	98%	99%	98%	98%	99%	98%	97%	99%	0.733
Birth at public hospital	3.7	83%	83%	81%	82%	81%	81%	81%	75%	81%	80%	**0.011**
Birth on Saturday/Sunday	0	27%	25%	28%	26%	27%	26%	26%	30%	24%	28%	0.682
Birth from 7:00 am to 6:59 pm	0.5	59%	60%	55%	57%	58%	58%	55%	63%	57%	57%	0.654
**Place and time of death**												
Death at the same municipality of birth	0	94%	94%	94%	91%	92%	91%	92%	93%	92%	93%	0.207
Death at hospital	0.02	96%	96%	94%	94%	99%	92%	98%	97%	97%	98%	**0.013**
Death at the same birth hospital	6.4	91%	90%	87%	87%	89%	86%	89%	89%	87%	90%	0.392
Death until 59 min after birth	0	12%	14%	10%	10%	10%	11%	12%	13%	12%	10%	0.805
**Maternal data**												
Maternal age < 20 years	0	22%	24%	22%	20%	19%	21%	18%	19%	18%	23%	**0.039**
Mother has a partner	5.1	42%	39%	37%	35%	33%	32%	32%	38%	49%	56%	**< 0.001**
Maternal schooling ≥8 years	2.5	60%	59%	61%	68%	69%	70%	74%	88%	73%	68%	**< 0.001**
Primiparity	8.1	38%	38%	39%	42%	21%	44%	47%	36%	43%	47%	**< 0.001**
Prenatal visits ≥4	2.9	74%	74%	74%	75%	75%	72%	73%	77%	72%	75%	0.944
Multiple gestation	0	7%	10%	8%	7%	8%	7%	10%	10%	7%	13%	**0.034**
Cesarean section	0.02	49%	50%	47%	51%	50%	51%	49%	54%	52%	53%	**0.019**
**Neonatal data**												
Gestational age 37–41 weeks	0	40%	39%	39%	39%	37%	39%	36%	37%	35%	31%	**< 0.001**
Birth weight ≥ 2500 g	03	39%	39%	36%	39%	35%	36%	35%	38%	37%	33%	**0.048**
Birth weight 500-1499 g	0.3	45%	48%	50%	45%	50%	50%	50%	50%	46%	52%	**0.021**
Male	0	53%	57%	55%	55%	53%	54%	59%	58%	59%	58%	**0.019**
Non-white	0.6	25%	28%	29%	27%	26%	29%	29%	30%	27%	25%	0.689
1st minute Apgar Score 0–3	8.3	70%	69%	66%	70%	67%	70%	67%	74%	73%	71%	0.050
5th minute Apgar Score 0–6	8.1	67%	65%	65%	66%	63%	68%	65%	70%	70%	66%	0.240

^a^ % of 6648 deaths without information for each variable #refers to chi-square test for linear trend

**Fig. 2:**
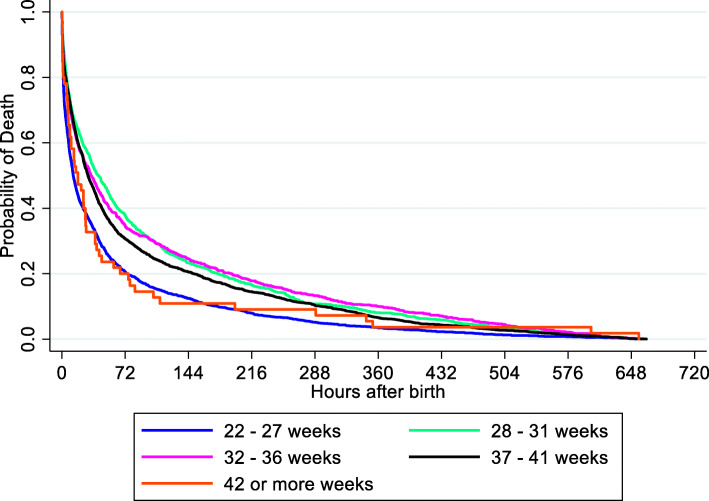
Age in hours at the time of death of the 6648 infants who died with perinatal asphyxia by Kaplan-Meier survival estimate according to the gestational age, São Paulo State, Brazil: 2004–2013

Figure 2 shows the Kaplan-Meier analysis of the age, in hours, at death. For all studied infants, median age until 50% of deaths occurred was 25.3 h (95%CI: 24.0; 27.2). For each GA group, the median age was: 12.8 h (95%CI: 11.8–14.4) for 22–27 weeks (2092 deaths); 41.1 (35.0–48.7) hours for 28–31 weeks (1038 deaths); 32.9 (27.4–39.1) hours for 32–36 weeks (979 deaths); 29.6 (27.4–32.5) hours for 37–41 weeks (2484 deaths).

Cox regression analysis of variables associated with neonatal death adjusted for year and region of death within São Paulo State, among all neonates who died with perinatal asphyxia, showed that the risk of death increased 16% if mothers had 0–3 prenatal care visits; 49 and 20% if the 1st minute Apgar score was 0/1 or 2/3, respectively, and 57% if death occurred at the same place of birth. On the other hand, among all the neonates who died with perinatal asphyxia in this study, the risk of death was reduced if the birth occurred in public hospitals (belonging to the Unified Health System – SUS) and if the infant was male. There was a significant interaction between mode of delivery and GA. Among all neonates who died with perinatal asphyxia, the risk of death increased for extremely premature neonates born by vaginal delivery and post-term infants born by cesarean section, but for neonates with GA of 28–36 weeks, the risk of death was reduced if they were born by cesarean section (Table 4).

**Table 4:** Epidemiological risk factors associated with deaths of infants up to 27 days after birth with perinatal asphyxia adjusted by year of death (Cox Regression), São Paulo State, Brazil: 2004–2013

	Hazard Ratio	95% CI
**Death at same place of birth**	1.57	1.43–1.7
**Birth at Unified Health System hospital**	0.92	0.86–0.99
**Number of antenatal care visits**		
0–3	1.16	1.07–1.25
4–6	1.05	0.98–1.12
**1st minute Apgar score**		
0–1	1.49	1.33–1.67
2–3	1.20	1.07–1.35
4–6	1.09	0.97–1.22
**Sex male**	0.90	0.86–0.95
**Gestational age x Delivery mode**	*p* = 0,006	
**37–41 weeks**		
Cesarean vs. Vaginal	1.01	0.93–1.11
**22–27 vs. 37–41 weeks**		
Vaginal	1.37	1.24–1.51
Cesarean	1.12	0.99–1.26
**28–31 vs. 37–41 weeks**		
Vaginal	0.89	0.79–1.02
Cesarean	0.77	0.69–0.87
**32–36 vs. 37–41 weeks**		
Vaginal	0.97	0.85–1.12
Cesarean	0.83	0.75–0.93
**≥ 42 vs. 37–41 weeks**		
Vaginal	1.10	0.69–1.76
Cesarean	1.91	1.27–2.87

## Discussion

A significant reduction of 32% in NMR with perinatal asphyxia over a 10-year period in São Paulo State was shown in this population-based study. The decrease in NMR with asphyxia started in 2008 and reached 0.95 deaths per 1000 live births in 2013, due mainly to the lower deaths in neonates with 32–36 weeks and ≥ 42 weeks of GA.

Globally the number of neonatal deaths declined to 2.4 million in 2019, with 17 deaths per thousand live births and approximately 6700 neonatal deaths every day, with the greatest risk of the youngest lives [15]. In this study, the decrease in NMR represents a reduction of 2.32 deaths per day in 2004 to 1.55 deaths in 2013. Although important, the rates presented in this study are much higher than neonatal deaths with birth asphyxia and birth trauma in high-income countries. Japan, for example had in 2015, a NMR of 0.92‰ and a NMR by birth asphyxia of 0.1‰ [16]. As a signatory country of the “Millennium Development Goals”, Brazil experienced a stable democracy and a moderate economic growth in the past decade, with gradual reduction in income inequalities and promotion of health care assistance by consolidation of the Unified Health System and expansion of family health strategy. With a special attention addressed to maternal and neonatal care, several policies were introduced, such as the “National Program for the Humanization of Pregnancy and Childbirth” (2000), the “Pact for the Reduction of Maternal and Newborn Mortality” (2004), the creation of local committees for the prevention of infant mortality (2005), the “Pact for Life” (2006), besides the “Stork Network” in 2011 [17]. The impact of these health care strategies may have contributed to the progressive reduction of neonatal deaths with perinatal asphyxia, mainly from 2008 onwards and first observed in neonates with 32 to 41 weeks of GA. The reduction verified in moderate preterm infants, followed by near term and term babies, throughout these years, demonstrates that the essential care of post-term, term and moderately preterm infants contributes to the prevention of avoidable deaths, with a great impact in public health [18]. Skilled maternal and immediate neonatal care may avoid 30–45% of deaths with asphyxia, and adequate neonatal resuscitation accounts for 5–20% of the reduction [19]. In this context, training of neonatal resuscitation skills in the studied region of the country probably contributed to the decrease in neonatal deaths with perinatal asphyxia. São Paulo State has the largest Neonatal Resuscitation Program (NRP) of the country, with 8665 physicians and 7155 health professionals trained until 2013 by the Brazilian Society of Pediatrics. The Brazilian NRP, updated according to the International Liaison Committee on Resuscitation Consensus on Science and Treatment Recommendation, contributes to the availability of standardized delivery room care to neonates at birth [20, 21].

In our study, very preterm and extremely preterm infants accounted for the most vulnerable group, with a more recent and smaller reduction of NMR with asphyxia. These births may point out to the fragilities of Brazilian health system that does not connect 43% of women to reference maternities for childbirth care, although this has been regulated in the country since 2007 [22]. The wandering of pregnant mothers in labor or at high risk to find a hospital vacancy leads to birth at low-complexity hospitals, as shown in the survey “Birth in Brazil”: 50% of babies weighing less than 1500 g died in hospitals without neonatal intensive care [23]. Neonatal intensive care units and highly skilled personnel are limited to large economic clusters distributed throughout São Paulo State. An inefficient regionalized perinatal care network impairs the transference of sick newborns to referral centers, leading to more deaths in the first day after birth, as shown in this study. The fact that around 90% of deaths in the present study occurred in the same hospital of birth may indicate that mechanisms of referral of sick neonates with multiple organ dysfunction due to perinatal asphyxia are not fully developed.

The median time until 50% of deaths occurred in the extremely preterm infants was 13 h, probably due to the high mortality rate in extremely low birth weight infants that receive cardiopulmonary resuscitation at birth [24]. Also, the limited chance of survival of these babies could interfere in the decision by Brazilian pediatricians to start or not resuscitation in delivery room [25]. The fact that vaginal delivery was associated with a higher chance of death with perinatal asphyxia reinforces that a decision not to invest in these extremely preterm infants may be associated with these fast deaths. On the other hand, the association of vaginal delivery and death with asphyxia in extremely preterm infants may be an indicator of the lack of a more complex neonatal care, which is highlighted by the association of higher risk of death for patients that birth and death occurred in the same hospital.

The group of 28 to 31 weeks had a median time of 41 h until 50% of deaths occurred, probably related to a variety of other causes besides intrapartum hypoxia that contributed to difficulties in the transition to the extra-uterine environment. These conditions require a well-equipped and staffed hospital, with the skills for intubation and replacement of surfactant in respiratory distress syndrome, and other intensive respiratory support, cardiovascular support if needed, early administration of antibiotics in the case of sepsis, feeding support or use of intravenous fluids [26], which is available only in centers with neonatal intensive care units throughout São Paulo State.

Unexpectedly, a shorter time until the occurrence of deaths was observed in moderate preterm (33 h) and term babies (29 h), compared to infants of 28–31 weeks. According to the series “Born too Soon”, many babies of both groups die needlessly for lack of simple and essential care, rather than lack of intensive care interventions [26]. Even though these two groups accounted for the largest reduction of neonatal mortality with asphyxia throughout the period, 52% of the 6648 neonatal deaths with perinatal asphyxia occurred in moderate preterm and term babies. Many factors could contribute to these early deaths, such as delay in attending pregnant women with obstetric emergencies or difficulties in recognizing the problem and delaying the decision to seek care, or even by problems in reach health care facilities that deliver quality care to neonates. During 2004 to 2013, the study revealed that 7 in 10 pregnant women whose neonates died with perinatal asphyxia attended more than 4 antenatal visits without the number of visits guaranteing the quality of prenatal care. The survey “Birth in Brazil” showed that, in the Southeast region of Brazil, where São Paulo State is located, the majority of the mothers had hand-held prenatal notes, but only 49% had received information about onset of labor, 61% about signs of risk during pregnancy, and 39% about activities to facilitate childbirth [27]. Therefore, the lack of information and the delay inf reaching a medical assistance center, due to a deficient hospital referral system for care during birth, may lead to unfavorable outcomes in Brazil, where most women seek hospital care by themselves and only one fifth is transported by ambulance [28]. As 25 and 18% of deaths of infants with birth weight, respectively, ≥2500 g and 1500–2499 g can be reduced by adequate care to mothers during childbirth [29], it is important to use our results to help to change the model of care in labor and birth. Improving quality of care through established protocols, monitoring service at facility-based care and creating proper mechanisms to transfer, besides allocating skilled manpower and finances to existing facilities, ensures a higher quality of care to mother and infant, reducing both neonatal and maternal deaths.

Number of antenatal visits, GA, delivery mode, 1st minute Apgar score, and death at the same place of birth were independently associated with a higher risk of death among infants that died with perinatal asphyxia until 27 days after birth in this study. As a key element of basic primary healthcare during pregnancy, antenatal care may prevent, detect, and treat risk factors early on, during pregnancy. A global study which included 616,347 mothers from low- and middle-income countries showed 3.1% of newborn deaths for mothers without antenatal care who were less educated and poorer, compared to 1.7% for those that attended at least one antenatal care visit [30]. In Brazil, in 2014, almost 35% of 641,264 pregnant women with inadequate prenatal visits had 4 or less years of schooling [31]. This is a vicious circle of poverty, lack of education, poor access to prenatal care and neonatal mortality, highlighted in our study by the association of inappropriate antenatal care and higher risk of neonatal death with asphyxia.

Intrapartum care is also a major component of the challenges that need to be addressed to decrease deaths associated with perinatal asphyxia. The finding that cesarean delivery was a protective variable against neonatal deaths with asphyxia in infants with 28–36 weeks shows that, possibly, access to adequate obstetric care was one of the bottlenecks of health assistance in the studied group. Difficulties to reach medical care, to be attended at the right time, lack of hospital beds or skilled professionals may delay the decision to interrupt the gestation during hypoxic-ischemic events that precede the full cascade of multi-organ compromise that characterize perinatal asphyxia. This may explain the presence of cesarean section as a protective variable in infants with 28–36 weeks of gestation and the vaginal delivery as a risk variable in infants with 22–27 weeks, among neonates that died with perinatal asphyxia.

The ideal number of neonatal intensive care beds is 4.0 per 1000 live births. Among the 3.6 neonatal intensive care beds per 1000 live births available in São Paulo State, only half belongs to the public health system, which reflects overloaded neonatal intensive care units that are unable to attend all high-risk births [32]. The lack of public intensive care beds may contribute to unattended babies dying at the same place of birth. To avert these preventable deaths there is no need to have a high complexity hospital throughout all 645 municipalities in São Paulo State, but a regionalized, hierarchical and integrated network that can link primary, secondary and third levels of maternal and neonatal assistance. In this context, it should be noted the lower risk of death at public hospitals in the present study. “Birth in Brazil”, a nationwide Brazilian cohort study revealed that public hospitals have higher quality than the private ones in the Southern region, in many aspects such as: 83% of the public healthcare facilities were teaching hospitals against 21% of the private ones; 56% high-risk referral hospitals were public hospitals with no private referral hospitals; and 94% of public hospitals had all the neonatal emergency equipment versus 76% of the private ones [33].

Due to biological advantages, female neonatal mortality is lower than male neonatal mortality. Excess female mortality may be an indicator of influences that outweigh the survival advantages of girls, including unfair distribution of resources, discrimination, unequal opportunities or different treatment for girls and boys [34]. Although Brazil is not among the countries with higher excess female infant mortality, we found that male sex was independently associated with a lower risk of death with perinatal asphyxia, which suggests that gender inequalities in neonatal health care should be better explored.

This is the first population-based study of neonatal deaths associated with perinatal asphyxia in São Paulo State with high coverage of information due to the linkage of Death Certificate to the correspondent Live Birth Certificate. Information on demographical and perinatal variables was 100% available for 20 variables except for “parity”, “Apgar score at first” and “fifth minutes”, with 92% of information. However, the use of secondary data based exclusively at information on Death and Live Birth Certificates prevents us from analyzing the process that led to perinatal asphyxia, relying only at the clinical reasoning of the physician who filled the Death Certificate. Nonetheless, studies like ours are the opportunity to count and follow up neonatal death so the real bottleneck of perinatal assistance can be shown, and specific maternal and neonatal public policies may be designed and implemented.

## Conclusion

Our study showed a significant reduction of NMR associated with perinatal asphyxia in this 10-year period, mainly in newborn infants with gestational age ≥ 32 weeks in São Paulo State, Brazil. The first day of life is the critical moment for these infants. Variables such as antenatal care, GA, delivery mode, infant’s gender and place of death were associated with risk of death among neonatal mortality with perinatal asphyxia. Public health policies should be planned to improve quality of perinatal care in all levels, along with a robust and organized referral system.

## Data Availability

The complete database is not publically available, but it available on request with the corresponding author after permission of Fundação SEADE.

## Abbreviations

MDG: Millennium Development Goals
NMR: Neonatal mortality rate;
HDI: Human Development Index
ICD: International Classification of Diseases
Fundação SEADE: Foundation of the State System of Data Analysis
GA: Gestational Age
NRP: Neonatal Resuscitation Program.
